# Melioration Learning in Two-Person Games

**DOI:** 10.1371/journal.pone.0166708

**Published:** 2016-11-16

**Authors:** Johannes Zschache

**Affiliations:** Institute of Sociology, Leipzig University, Leipzig, Germany; Peking University, CHINA

## Abstract

Melioration learning is an empirically well-grounded model of reinforcement learning. By means of computer simulations, this paper derives predictions for several repeatedly played two-person games from this model. The results indicate a likely convergence to a pure Nash equilibrium of the game. If no pure equilibrium exists, the relative frequencies of choice may approach the predictions of the mixed Nash equilibrium. Yet in some games, no stable state is reached.

## Introduction

Various learning models have been analysed in the game-theoretic literature. The best known ones, such as fictitious play or Bayesian learning, describe normative processes that enable the players to find an equilibrium during the repeated play of a game [1]. Those models presume that information about the preferences and past actions of all players is available. More recently, researchers have evaluated whether equilibria can be reached without knowing the preferences of other players [2] or even without considering the other players’ presence [3]. The latter condition was called *radically* or *completely uncoupled* learning.

In completely uncoupled learning, a player’s strategy is based only on his own previous actions and rewards. Some dynamics still ensure the convergence to Nash *ε*-equilibria or pure Nash equilibria [4]. More specifically, *regret-testing* [3, 5] and *interactive trial-and-error (ITE) learning* [6] are two examples of completely uncoupled learning that imply this convergence.

Under the name of *reinforcement learning*, further completely uncoupled dynamics have been analysed in different fields. For instance in economics, one of these models stems from Roth and Erev [7]. In computer sciences, multiple studies in artificial intelligence deal with algorithms of reinforcement learning, e.g. *Q-learning* or *SARSA* [8]. Also some psychological models are entirely based on own experiences [9] and, hence, completely uncoupled.

In contrast to regret-testing or ITE learning, most models of reinforcement learning are not guaranteed to converge to an equilibrium in interactive situations. Instead of being designed to imply this convergence, they constitute simple and realistic representations of human learning. In particular psychological models have been built to represent the development of human behaviour as realistic as possible while keeping it analytically tractable, e.g. [10].

This paper strives for the usage of a simple psychological model of completely uncoupled learning. It is called *melioration learning* and may not converge towards equilibrium states. The next section describes the underlying theory of decision-making and its implementation as instance of the Q-learning algorithm. Afterwards, the model is applied to various two-person games. A connection to the previous literature is established by comparing its predictions to the outcomes of the Roth-Erev model [7].

## Melioration learning

Established by Herrnstein and Vaughan [11], *melioration learning* is a theory of individual decision-making from behavioural psychology. It was introduced as explanation of the *matching law* [12], which describes an often observed regularity of individual behaviour [13–23]. In the past, many empirical studies have validated the predictions of melioration learning [24–31].

Generally speaking, melioration learning states that behaviour is strengthened by highly valued events that are perceived as consequences of this behaviour. In the original literature, this process was phrased as “behaviour shifts toward higher local rates of reinforcement” (p. 75, [12]). The *local reinforcement rate* was defined as “the reinforcement actually obtained from an alternative [.] divided by the time allocated to it” (p. 76, [12]).

Elsewhere, Vaughan and Herrnstein [26] more formally described the process of melioration by a differential equation. Let there be a two-element choice set {1, 2}. Given a point in time *t* ∈ (0, ∞), *p*_*i*_(*t*) ∈ [0, 1] denotes the relative frequency of having chosen alternative *i* ∈ {1, 2}. The authors stated that the frequency *p*_1_(*t*) changes over time in accordance with

$$\frac{dp_{1}\left(t\right)}{dt}=f\left({\hat{v}}_{1}\left(t\right)-{\hat{v}}_{2}\left(t\right)\right).$$ (1)

In Eq (1), f:R→R is a differentiable and strictly monotonically increasing function with *f*(0) = 0. The term ${\hat{v}}_{i}\left(t\right)$ (*i* ∈ {1, 2}) stands for the local reinforcement rate of alternative *i* at time *t*.

Without specifying the function *f* of Eq (1), the melioration learning rule remains vague, and long-term behaviour cannot be analysed. In contrast to previous specifications [32–34], this paper presents a formal representation of melioration learning that is perfectly consistent with Eq (1) and builds on a well-established algorithm of reinforcement learning. More precisely, melioration is suggested to be formalised by an instance of the *Q-learning* algorithm [35] with *ε-greedy* strategy.

**Fig 1:**
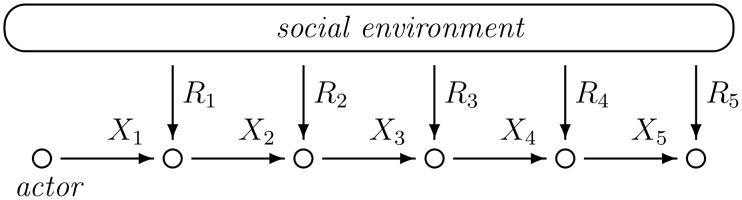
The situation of sequential decision-making.

Q-learning is a form of temporal-difference (TD) learning and originates from a sub-field of artificial intelligence [8]. While TD models were initially used to represent classical conditioning [36], they can be “applied to stochastic sequential decision tasks to produce an analog of instrumental learning” (pp. 541-542, [37]). A general model of *sequential decision tasks* is specified in Definition 1 and illustrated in Fig 1.

**Definition 1** Let *E* be a finite set of choice alternatives. A **situation of sequential decision-making** is given by two stochastic processes ${\left(X_{t}\right)}_{t=1}^{\infty }$ and ${\left(R_{t}\right)}_{t=1}^{\infty }$ with values in *E* and [0, ∞), respectively.

In the situation of Definition 1, decisions are made in discrete time steps t∈N. At time *t*, the actor emits an action by choosing an element *X*_*t*_ ∈ *E* from the set of alternatives. Subsequently, a non-negative *reward* *R*_*t*_ is received from the *social environment*. In this paper, the action-process ${\left(X_{t}\right)}_{t=1}^{\infty }$ is specified by Algorithm 1, which contains an instance of Q-learning with *ε*-greedy strategy.

**Algorithm 1** The melioration learning algorithm

**Require:** exploration rate *ε* ∈ (0, 1), set of alternatives *E*

1: *t* ← 0

2: initialise *Q*_1_(*j*)←0, for all *j* ∈ *E*

3: initialise *K*_1_(*j*)←0, for all *j* ∈ *E*

4: **repeat**

5:  *t* ← *t* + 1

6:  *r* ← random number between 0 and 1 (uniformly distributed)

7:  **if** *ε* > *r* **then**

8:   choose a random action *X*_*t*_ ← *e* ∈ *E* using a uniform distribution

9:  **else**

10:   choose an action *X*_*t*_ ← *e* such that *Q*_*t*_(*e*) = max_*j* ∈ *E*_ *Q*_*t*_(*j*)

11:  **end if**

12:  observe reward *R*_*t*_ = *y*

13:  *K*_*t*+1_(*e*) ← *K*_*t*_(*e*) + 1

14:  $Q_{t+1}\left(e\right)\leftarrow Q_{t}\left(e\right)+\frac{1}{K_{t+1}\left(e\right)}\left(y-Q_{t}\left(e\right)\right)$

15:  **for all** *j* ≠ *e* **do**

16:   *K*_*t*+1_(*j*) ← *K*_*t*_(*j*)

17:   *Q*_*t*+1_(*j*) ← *Q*_*t*_(*j*)

18:  **end for**

19: **until** termination

In Algorithm 1, an actor is assumed to maintain a set of *Q-values* {*Q*_*t*_(*e*)}_*e* ∈ *E*_ at every time step t∈N. The Q-values are initially set to zero and iteratively updated. At every round, an alternative *e* ∈ *E* is chosen randomly with probability *ε* or greedily otherwise. Greedy choice means that an alternative with the currently highest Q-value is selected. The Q-value *Q*_*t*_(*e*) of the chosen alternative *e* is modified by the realisation of *R*_*t*_ such that it equals the average of all past rewards of *e*.

In the words of Herrnstein and Vaughan [11], *Q*_*t*_(*e*) corresponds to the local reinforcement rate of action *e* ∈ *E* at time t∈N. If the actor always chooses an action with the currently highest Q-value, the relative frequency of this action increases as required by Eq (1). Consequently, Algorithm 1 with *ε* = 0 conforms to the theory of melioration learning. A strictly positive exploration rate *ε* > 0 allows a trade-off between exploiting the currently best actions and exploring alternatives. If this rate decreases sufficiently slowly towards zero over time, past research proved that Q-learning converges to optimal behaviour under certain assumptions of stationarity [38, 39]. For example, convergence is assured if, for every t∈N, the reward *R*_*t*_ is bounded and its expected value depends only on *X*_*t*_.

However, convergence of Q-learning is impeded if multiple persons interact and reinforcements are contingent upon the decisions of everyone (p. 451, [40]). While equilibria are reached in some instances of the prisoner’s dilemma or the coordination game [41–43], the behaviour fails to converge in others. The results depend on the reward structure of the situation [44] as well as the particular version of Q-learning [45].

In the next section, various examples of two-person games are explored by agent-based simulations. The outcomes of Algorithm 1 are compared to the predictions of another model of reinforcement learning, which is widely known in economics and was developed by Roth and Erev [7]. Algorithm 2 specifies this model. Similar to Algorithm 1, an actor holds a set of values {*P*_*t*_(*e*)}_*e* ∈ *E*_ that reflect the previous experiences with the alternatives. In [7], these values are called *propensities*. At each time step, an alternative *e* ∈ *E* is chosen with probability $\frac{P_{t}\left(e\right)}{\sum_{j\in E}P_{t}\left(j\right)}$. The parameter *ε* maintains a level of exploration.

**Algorithm 2** The Roth-Erev learning algorithm

**Require:** exploration rate *ε* ∈ (0, 1), set of alternatives *E*

1: *t* ← 0

2: initialise *P*_1_(*e*) ← 1, for all *e* ∈ *E*

3: **repeat**

4:  *t* ← *t* + 1

5:  choose action *X*_*t*_ ← *e* ∈ *E* randomly using the probabilities ${\left\{\frac{P_{t}\left(e\right)}{\sum_{j\in E}P_{t}\left(j\right)}\right\}}_{e\in E}$

6:  observe reward *R*_*t*_ = *y*

7:  *P*_*t*+1_(*e*) ← *P*_*t*_(*e*) + (1 − *ε*)*y*

8:  **for all** *j* ≠ *e* **do**

9:   $P_{t+1}\left(j\right)\leftarrow P_{t}\left(j\right)+\frac{\varepsilon }{|E|-1}y$

10:  **end for**

11: **until** termination

There are two small differences between Algorithm 2 and the original model of [7]. First, *gradual forgetting* is not considered because the melioration algorithm omits this feature as well. Second, the exploration quantity $\frac{\varepsilon }{|E|-1}y$ is added to all alternatives instead of just the “adjacent” ones. In [46], this approach was used for two-action games or if a linear order of the alternatives was absent.

The following analysis focuses on the Roth-Erev model instead of other learning processes because it is similar to melioration. Both models take a “mechanistic perspective on learning”, which means that “people are assumed to learn according to fixed mechanisms or routines” (p. 903, [47]). Additionally, simple versions with only one parameter (the exploration rate) exist. Other models of reinforcement learning, such as regret-testing, ITE, Bush-Mosteller [48], or experience-weighted attraction [49], require additional assumptions and the specification of further parameters.

## Results

Algorithms 1 and 2 were applied to different two-person games by means of agent-based simulations. The simulations were implemented in NetLogo [50]. All games are presented in normal-form. The two players, which are also called *agents*, are labelled by “x” and “y”. Capitalised letters or integers depict the alternatives. The following rules specify the simulations.

- For each game, a simulation of 20000 pairs of agents was run. Every agent interacted with the same partner during the whole simulation.
- Half of the pairs of agents employed Algorithm 1 (melioration learning). The other half used Algorithm 2 (Roth-Erev). In both cases, *ε* was set to 0.1.
- Every player repeatedly chose one of the alternatives according to Algorithm 1 or 2 until 1000 choices had been made.
- The agents observed only their own choices and rewards. They were not aware of the structure of the game or the partner’s choices and rewards.
- The payoff matrices show mean rewards. The actual rewards were drawn from normal distributions with standard deviations of one.

**Fig 2:**
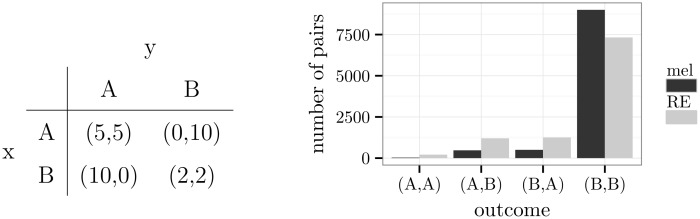
A prisoner’s dilemma and simulation results.

Statistical tests were omitted in the comparison of the two learning models because they are largely unnecessary. Since there were 10000 pairs of agents in each group, any standard test would have marked a difference as low as 150 pairs as statistically significant. For example, in the histogram of Fig 2, the first two bars at (*A*,*A*) show a difference of 178 pairs. The reader may decide whether the reported differences in numbers are theoretical or practical *significant*.

In the following, three classes of two-person games are distinguished. The first class contains games in which both players have a (weakly) dominant alternative. Second, games without dominant alternatives but with several pure Nash equilibria are considered. The last class covers games with exactly one mixed Nash equilibrium. This division is not exhaustive, but it clarifies the properties of melioration learning in two-person games.

### Games with dominant alternatives

An alternative of a player is *dominant* if the choice of this alternative comes with a mean reward that is strictly greater than the mean reward of any other alternative given one choice of the partner and greater than or equal to the mean reward of any other alternative given the other choices of the partner (cf. *weak dominance* in [51], p. 77). A representative member of this class of games is the *prisoner’s dilemma*. In the example of Fig 2, alternative *B* is dominant for both players. The outcome (*B*,*B*) is, therefore, a Nash equilibrium. All other outcomes are optimal.

In Fig 2, the frequency distribution of pairs of agents at the 1000th round of the simulation is shown (for the temporal development, see S1 Fig). It is distinguished between pairs of agents who learned by melioration (mel) and pairs of agents who used the Roth-Erev model (RE). Both types of agents predominantly chose the Nash equilibrium. Because of the exploration rate, also the non-equilibrium outcomes (*A*,*B*) and (*B*,*A*) occurred. In case of melioration learning, the frequencies approximated the expected ones: $10\;000\cdot \frac{\varepsilon }{2}\cdot \left(1-\frac{\varepsilon }{2}\right)=475$. Agents who used the Roth-Erev model showed slightly higher frequencies of non-equilibrium outcomes.

**Fig 3:**
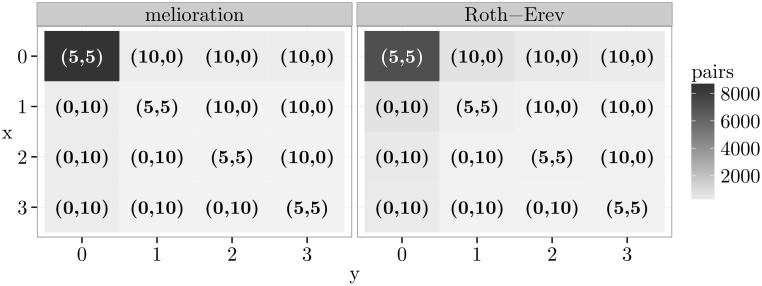
The game “guess $\frac{2}{3}$ of the average” and simulation results.

Another example of a game with dominant alternative is called “guess $\frac{2}{3}$ of the average”. Fig 3 contains a discrete version of this game with four alternatives. In this game, each player tries to guess what two-thirds of the average of both guesses will be. The agent who is closest to this value “wins” the game. In the particular example of Fig 3, one can choose an integer between 0 and 3. The choice of alternative 0 is dominant. The reward table and the simulation results are displayed in the same plot by heat maps. The background colour of a cell is light grey if only few pairs of agents chose this outcome at the 1 000th round of the simulation. It is close to black if many pairs did so. The heat maps show that almost all agents learned to choose the dominant alternative 0, which constitutes the only Nash equilibrium (see also S2 Fig).

**Fig 4:**
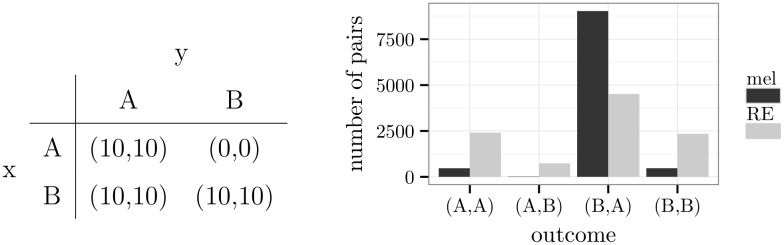
A game with three optimal Nash equilibria.

While both models implied a tendency towards the dominant alternative, the Roth-Erev model ended up slightly more often in outcomes with dominated alternatives. This effect was more clearly seen in the game of Fig 4, in which alternative B is dominant for player x, and alternative A is dominant for player y. Hence, the outcome (*B*,*A*) is a Nash equilibrium. Additionally, (*A*,*A*) and (*B*,*B*) are Nash equilibria, which are not *payoff-dominated* by (*B*,*A*) because they involve the same mean rewards (p. 81, [52]). The simulations revealed that all agents preferred the first equilibrium (*B*,*A*) instead of (*A*,*A*) and (*B*,*B*). But the Roth-Erev model maintained a relatively high probability of choosing the dominated alternative. This probability did not decrease with further rounds of the simulation (see S3 Fig).

In case of melioration learning, the acquisition of the dominant alternative was due to the exploration rate. Exploration guaranteed that the fourth outcome (*A*,*B*) was selected occasionally, especially in the beginning of the simulation. For player x, this meant that the average value of alternative *A* (*Q*_*t*_(*A*)) was between 0 and 10. The Q-value of alternative *B*, on the other hand, was approximately 10. The reverse held for player y, which led to the combination (*B*,*A*) in rounds without exploration.

**Result 1** In two-person games, the process of melioration learning yielded the choice of a (weakly) dominant alternative.

### Games with multiple pure equilibria

The exploration rate was a key factor in the simulations of the previous section because it rendered dominated alternatives inferior. In games without dominant alternative, this argument did not apply, and actors were not drawn to a single alternative. Games with a strictly mixed Nash equilibrium are considered in the next section. In this section, games with at least two pure equilibria are analysed.

**Fig 5:**
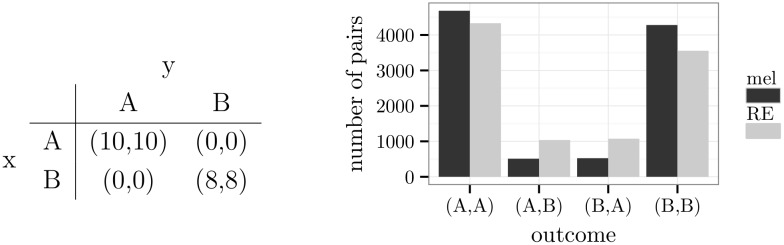
A coordination game and simulation results.

A basic game with two or more Nash equilibria is the *coordination game*. It refers to a class of situations in which the players prefer to coordinate their choices in some way. In the particular example of Fig 5, the outcomes (*A*,*A*) and (*B*,*B*) are pure Nash equilibria, and (*A*,*A*) payoff-dominates (*B*,*B*) because of higher mean rewards (p. 81, [52]). This game has an additional mixed equilibrium with probabilities $\left(A:\frac{4}{9},B:\frac{5}{9}\right)$ for both players.

At the 1000th round of the simulation, the agents chose mainly a pure Nash equilibrium and the payoff-dominant one with a slightly higher frequency. In other words, most pairs of agents were able to coordinate their choices. The deviations to (*A*,*B*) and (*B*,*A*) were due to the exploration rate and, similar to the previous simulations, more pronounced in case of the Roth-Erev model.

**Fig 6:**
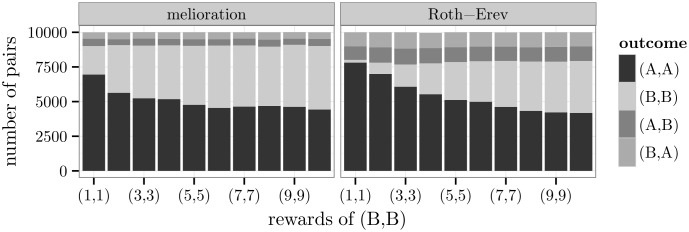
Relationship between the rewards of (*B*,*B*) and frequencies.

Further simulations revealed that the particular reward structure affected the distribution of agents among the two Nash equilibria. In particular, the frequency of the suboptimal equilibrium (*B*,*B*) depended on its expected rewards. As seen in Fig 6, the higher its rewards, the higher was its frequency (see also S4 Fig).

**Fig 7:**
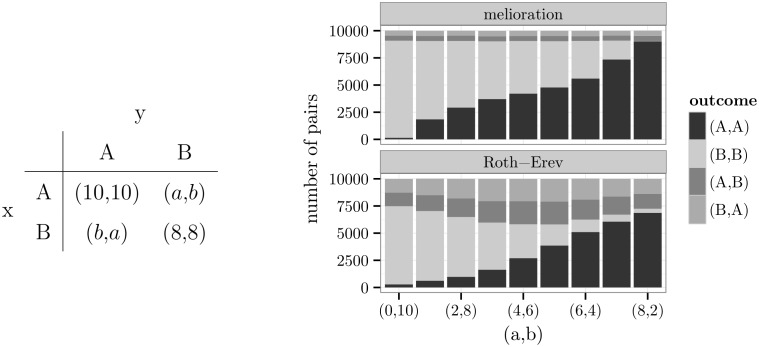
Relationship between non-equilibrium rewards and frequencies.

The distribution also changed with the rewards of the non-equilibrium outcomes (*A*,*B*) and (*B*,*A*). In the game of Fig 7, these rewards are set by two parameters *a* and *b*. Depending on the difference *b* − *a*, the agents were more strongly drawn to either (*A*,*A*) or (*B*,*B*). If *a* = 0 and *b* = 10, almost all pairs of agents chose (*B*,*B*). The number of pairs at (*B*,*B*) decreased with the difference *b* − *a* (see also S5 Fig).

This correlation can be explained when considering the melioration algorithm. The agents attached values *Q*_*t*_(*A*) and *Q*_*t*_(*B*) to alternative A and B irrespective of the choice of the other agent. Because of the exploration rate, also the outcomes (*A*,*B*) and (*B*,*A*) emerged occasionally. This means that the value of action A increased with the reward *a* and the value *Q*_*t*_(*B*) with *b*. Therefore, the tendency to choose (*A*,*A*) instead of (*B*,*B*) grew if *a* was raised or *b* was lowered.

The results of Fig 7 reflect a tendency towards the *maximin* alternative. An alternative is maximin if its choice leads to a maximum of all rewards that are minimal over the choices of the partner (p. 72, [51]). With *a* = 0 and *b* = 10, B was the maximin alternative for both players. On the contrary, A was their maximin alternative if *a* = 8 and *b* = 2. In the case of *a* = *b* = 5, both alternatives were maximin. Because the latter implied an indifference between the alternatives, all four outcomes should have occurred with the same frequency. For the Roth-Erev model, this was approximately correct. But agents who learned by melioration still coordinated their actions, and slightly more agents ended up in (*A*,*A*) (9.567 pairs) than in (*B*,*B*) (8.498 pairs).

**Fig 8:**
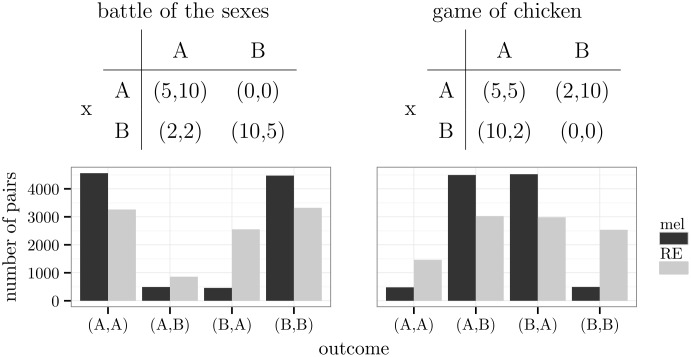
A “battle of the sexes” and a game of chicken.

In comparison, the melioration model was more successful in the coordination of actions than the Roth-Erev model. The latter led to non-equilibrium outcomes more frequently than predicted by the exploration rate. This was even more apparent in the “battle of the sexes”, which is a particular kind of coordination game. It describes an interaction between two persons with complementary preferences about two alternatives but with an additional preference for choosing the same one. A sample reward matrix is given by the left-sided table of Fig 8. There are two pure and one mixed Nash equilibria: (*A*,*A*); (*B*,*B*); $\left(x:(A:\frac{3}{13},B:\frac{10}{13}),y:(A:\frac{10}{13},B:\frac{3}{13})\right)$. Both pure equilibria are optimal. The outcome (*B*,*A*) consists of the maximin alternatives.

The simulations showed that most pairs of meliorating agents wound up in (*A*,*A*) or (*B*,*B*) (see also S6 Fig). Because of the symmetry of the game, there is no criterion that favours one of the two pure equilibria. Harsanyi [53] called this state *bargaining deadlock* between (*A*,*A*) and (*B*,*B*). While Harsanyi suggested the third (mixed) equilibrium as solution to the game (p. 279, [53]), simulations of melioration learning yielded an equal division of the pairs. If agents used the Roth-Erev model, also the suboptimal maximin outcome (*B*,*A*) appeared frequently.

A similar effect arose in the *game of chicken* (right-sided table of Fig 8), which resembles a basic conflict between two parties that requires the retreat of at least one of them to be solved. In this case, agents who learned by melioration predominantly chose one of the two pure Nash equilibria: (*A*,*B*) or (*B*,*A*). The Roth-Erev model implied the regular choice of the worst outcome (*B*,*B*) (see also S7 Fig).

**Fig 9:**
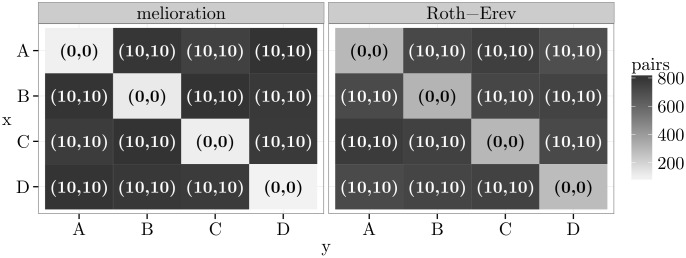
A dispersion game and simulation results.

Finally, a game with more than two pure Nash equilibria was analysed. Fig 9 contains heat maps of a *dispersion game* with four alternatives. It is, in some respect, the opposite of a coordination game. Each agent prefers not to match the choice of the other agent. This means that all but the diagonal outcomes are optimal Nash equilibria. Consequently, most agents of the simulations were distributed evenly among the non-diagonal outcomes. Agents who applied the Roth-Erev model were more often found in non-equilibrium outcomes (see also S8 Fig).

**Result 2** In two-person games without dominant alternatives, agents who learned by melioration arrived at one of the pure Nash equilibria. The frequency distribution over the equilibria depended on the structure of the game.

### Games without pure Nash equilibria

Simulations of games without pure equilibria required a higher number of rounds until the behaviour of the agents had converged. Therefore, the following simulations were run with only 2000 pairs of agents but for 20000 rounds of the game. The relative frequencies of choice were calculated for the whole period of 20000 rounds and for each agent separately. Furthermore, a slightly higher exploration rate (*ε* = 0.2) was assumed because it supported the speed of convergence (see S9 Fig).

**Fig 10:**
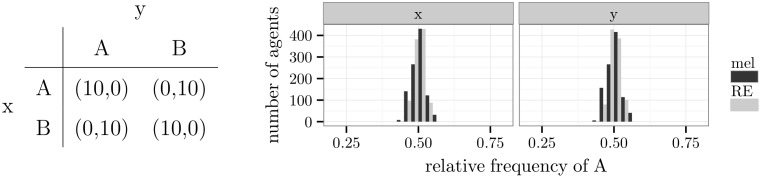
The game “matching pennies” and simulation results.

First, the game “matching pennies” as shown in Fig 10 was analysed. It is a zero-sum game, and its single Nash equilibrium is given by the probabilities (*A*: 0.5,*B*: 0.5) for both players. Fig 10 contains histograms over the relative frequencies of alternative *A*. For both types of players, the relative frequencies were in accordance with the probabilities of the mixed Nash equilibrium. The agents displayed a mix of alternatives in which each was chosen half of the time.

**Fig 11:**
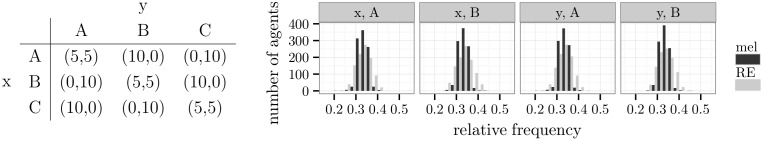
The game “rock-paper-scissors” and simulation results.

A similar result was obtained in the game “rock-paper-scissors”, which is zero-sum with three alternatives per player (Fig 11). The agents’ behaviour approached the predictions of the mixed Nash equilibrium: $\left(A:\frac{1}{3},B:\frac{1}{3},C:\frac{1}{3}\right)$. The rate of convergence is seen in S10 Fig.

**Fig 12:**
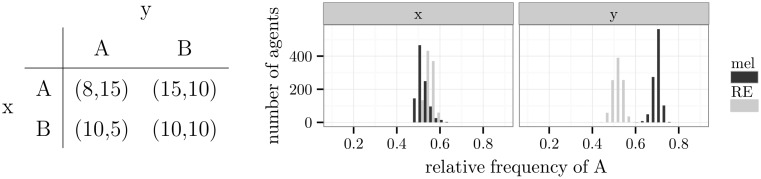
An example of the inspection game and simulation results.

Fig 12 displays a game that is not zero-sum and has a single mixed Nash equilibrium at $\left(x:(A:\frac{1}{2},B:\frac{1}{2}),y:(A:\frac{5}{7},B:\frac{2}{7})\right)$. In the past, this game was taken to model the interaction between criminals and police [54] and was, therefore, called *inspection game* [55]. The criminal (player x) chooses between committing a crime (*A*) or no crime (*B*). The inspector (player y) either inspects the criminal (*A*) or spares him (*B*). Committing a crime is beneficial if and only if no inspection takes place. An inspection is rewarding if and only if a crime occurs.

The simulation demonstrated that the behaviour of agents who learned by melioration approached the Nash equilibrium (see also S11 Fig). Further simulations were run with different payoffs for player x given the outcome (*A*,*A*). This payoff refers to the punishment of a crime. Since the predictions of the Nash equilibrium for player x remained constant and the results of the simulations stayed in line with the Nash equilibrium, criminals who learned by melioration chose to commit a crime with a relative frequency of 0.5 regardless of the level of punishment.

**Fig 13:**
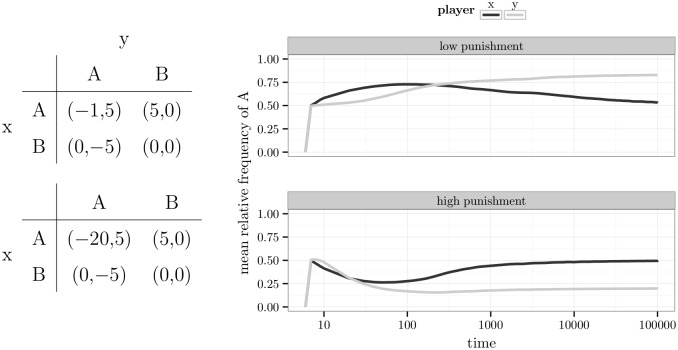
The inspection game with low or high punishment.

In previous research, laboratory experiments indicated that the level of punishment has an effect on the crime rate. More specifically, the level of punishment was negatively correlated with the crime rate [55]. However, the experiments lasted for only 15 rounds of decision-making. If humans learn slowly, the behaviour might have not converged to a stable point yet. In Fig 13, the temporal development of the relative frequencies of committing a crime are shown for the two games in [55]. All agents used the melioration learning model. The mean value of 1000 agents is plotted on a logarithmic scale of time. In case of low punishment (upper row), the Nash equilibrium (0.5) was approached from above. If punishment was high (lower row), the equilibrium was approached from below. Hence, there was a long period in which crime rates were higher for low punishment than for high punishment. Also the inspection rates conformed qualitatively to the experimental results if it is focused on early rounds.

**Fig 14:**
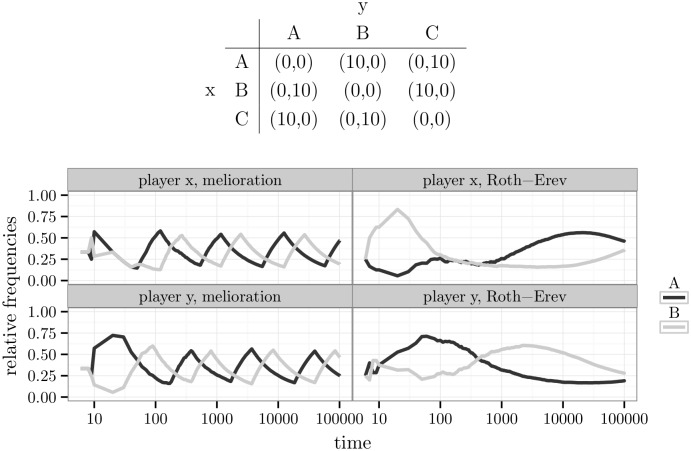
Shapley’s game and simulation results.

Last, some games impeded the convergence of the behaviour of agents who learned by melioration or the Roth-Erev model. One example is presented in Fig 14. This game was sometimes referred to as *Shapley’s game* and known for its difficulties in regard to the convergence of learning algorithms [56]. It is similar to the game “rock-paper-scissors” except for the diagonal rewards, which are (0, 0) instead of (5, 5). The Nash equilibrium is given by $\left(A:\frac{1}{3},B:\frac{1}{3},C:\frac{1}{3}\right)$.

The plots of Fig 14 depict the changes in relative frequencies of two particular players. If agents learned by melioration, the relative frequencies of all three alternatives rose and fell in sequence without any clear tendency towards convergence. This implied a constant change in outcomes: from (B,A) to (C,A) to (C,B) to (A,B) to (A,C) to (B,C) and back to (B,A). Since the time is on logarithmic scale, the lengths of the waves increased with time. But there was no decrease in the height of the waves, which could have led to a stable outcome. In case of the Roth-Erev model, the dynamic was slower, but no convergence was visible as well.

**Result 3** In two-person games without pure Nash equilibrium, agents who learned by melioration chose several alternatives with strictly positive relative frequency. In some of the games, the long-term relative frequencies corresponded to the mixed Nash equilibrium. Other games prevented the convergence of the agents’ behaviour.

## Conclusion

A simple process of completely uncoupled learning was investigated. It differs from previous models such as regret-testing or trial-and-error learning because, on the one hand, it is derived from empirical research and, on the other hand, the convergence to equilibrium states in social interactions is not guaranteed.

Nevertheless, computer simulations revealed that the outcomes of melioration were largely in line with game-theoretical predictions. More specifically, actors who learned by melioration chose a dominant alternative in two-person games. If no alternative was dominant, mainly pure Nash equilibria occurred. The structure of the game, which includes the rewards of non-equilibria, affected the distribution of outcomes. Compared to the Roth-Erev model, pure equilibria were selected with a higher frequency, and the melioration model was more successful in the selection of optimal ones.

In contrast to earlier models of learning, very few assumptions about available information and cognitive skills are needed. The actors must remember their own choices, observe the subsequent rewards, and be able to aggregate them to average values. They can neglect the other actors, their decisions and outcomes. Furthermore, apart from the exploration rate, the decisions are deterministic. No probabilities of choice and stochastically independent decisions are required.

In the past, melioration was often seen as too simplistic to adequately represent the complexity of human behaviour [57]. Yet, its predictions might be sufficiently accurate on a social level. Another advantage of melioration learning is that, with Q-learning, there is an algorithm that implements this theory and has been extensively studied in the past. First, this means that results about its convergence can be appropriated for an application in social theory. Second, multiple extensions of Q-learning exist. If melioration turns out to be too simple, there are many ways to adjust the model in order to be a more realistic representation of human behaviour.

## Supporting Information

S1 Fig Temporal development of behaviour in the prisoner’s dilemma. The rate of the dominant alternative in the game of Fig 2.
(TIFF)

S2 FigTemporal development of behaviour in the game “guess $\frac{2}{3}$ of the average”.The rate of the dominant alternative in the game of Fig 3.(TIFF)

S3 Fig Temporal development of behaviour in the game with three optimal Nash equilibria. The rate of the dominant alternative in the game of Fig 4.
(TIFF)

S4 Fig Temporal development of behaviour in the coordination game. The rates of the outcomes (*A*,*A*) and (*B*,*B*) in the game of Fig 5 with different rewards for (*B*,*B*).
(TIFF)

S5 Fig Temporal development of behaviour in the coordination game. The rates of the outcomes (*A*,*A*) and (*B*,*B*) in the game of Fig 7 with different rewards (*a*,*b*).
(TIFF)

S6 Fig Temporal development of behaviour in the “battle of sexes”. The rate of outcome (*A*,*A*) or (*B*,*B*) in the first game of Fig 8.
(TIFF)

S7 Fig Temporal development of behaviour in the game of chicken. The rate of outcome (*A*,*B*) or (*B*,*A*) in the second game of Fig 8.
(TIFF)

S8 Fig Temporal development of behaviour in the dispersion game. The rate of pure Nash equilibria in the game of Fig 9.
(TIFF)

S9 Fig Temporal development of behaviour in the game “matching pennies”. The mean relative frequency of alternative *A* in the game of Fig 10. The ribbon indicates the standard deviation. The relative frequencies at a time *t* were calculated for the period from the start of the simulation until time point *t*.
(TIFF)

S10 Fig Temporal development of behaviour in the game “rock-paper-scissors”. The mean relative frequency of alternative *A* in the game of Fig 11. The ribbon indicates the standard deviation. The relative frequencies at a time *t* were calculated for the period from the start of the simulation until time point *t*.
(TIFF)

S11 Fig Temporal development of behaviour in the inspection game. The mean relative frequency of alternative *A* in the game of Fig 12. The ribbon indicates the standard deviation. The relative frequencies at a time *t* were calculated for the period from the start of the simulation until time point *t*.
(TIFF)
