# Supplementary figures and images for: Melioration Learning in Two-Person Games
Source: PLoS One. 2016 Nov 16;11(11):e0166708. doi: 10.1371/journal.pone.0166708 (PMC5112854; doi:10.1371/journal.pone.0166708)

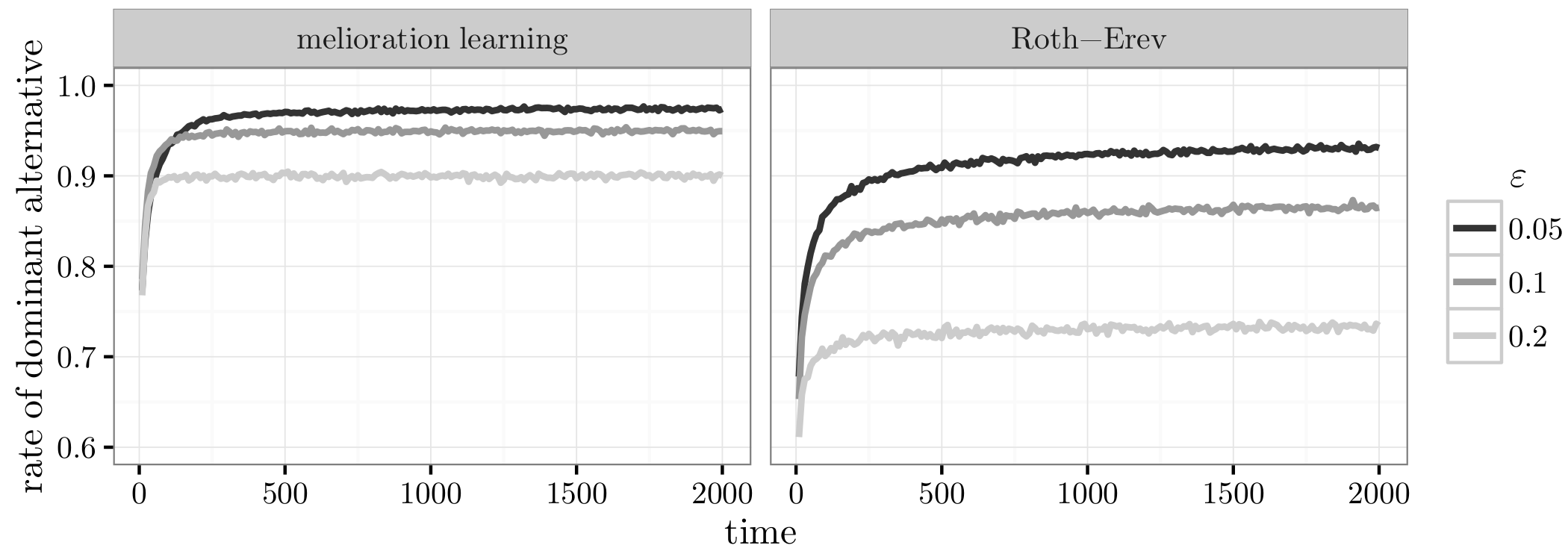

Supplement: S1 Fig — The rate of the dominant alternative in the game of Fig 2. (TIFF) [file pone.0166708.s001.tiff]

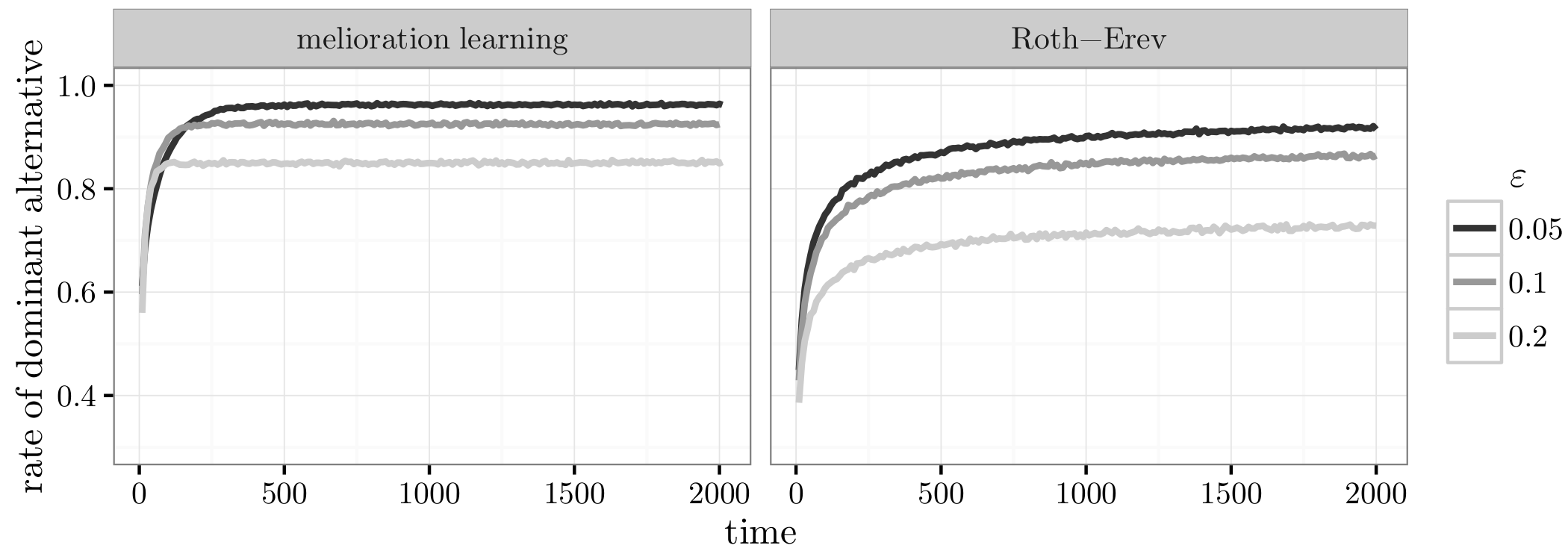

Supplement: S2 Fig — The rate of the dominant alternative in the game of Fig 3. (TIFF) [file pone.0166708.s002.tiff]

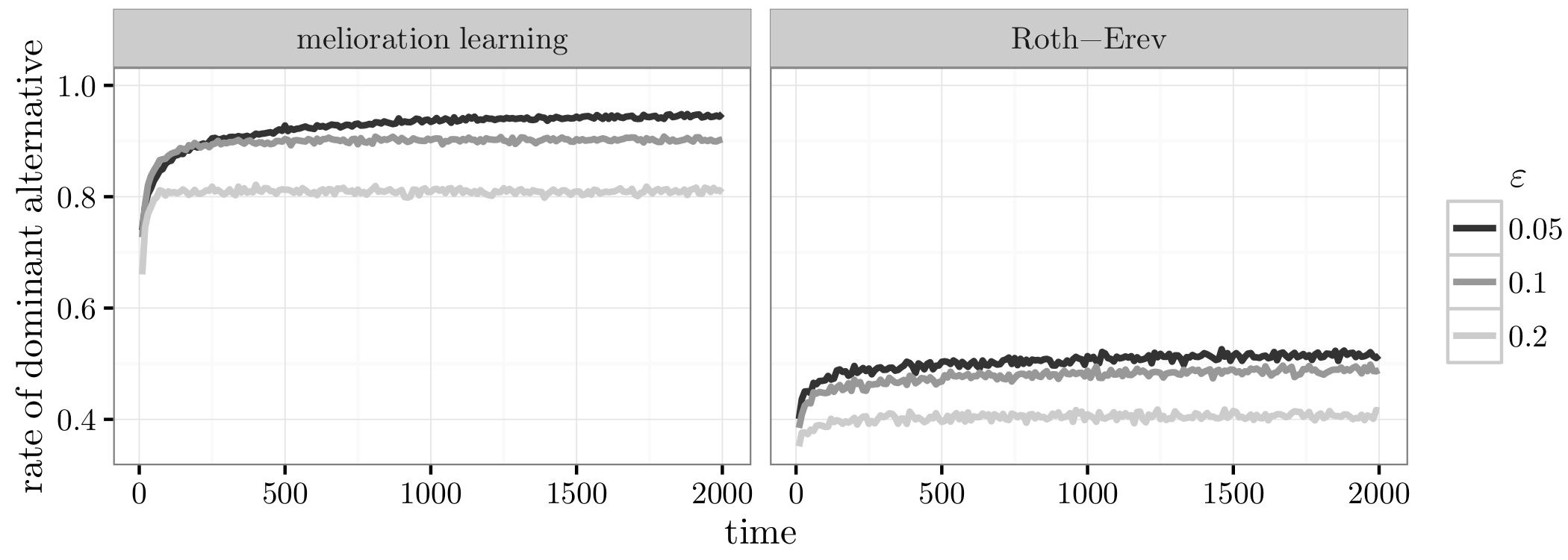

Supplement: S3 Fig — The rate of the dominant alternative in the game of Fig 4. (TIFF) [file pone.0166708.s003.tiff]

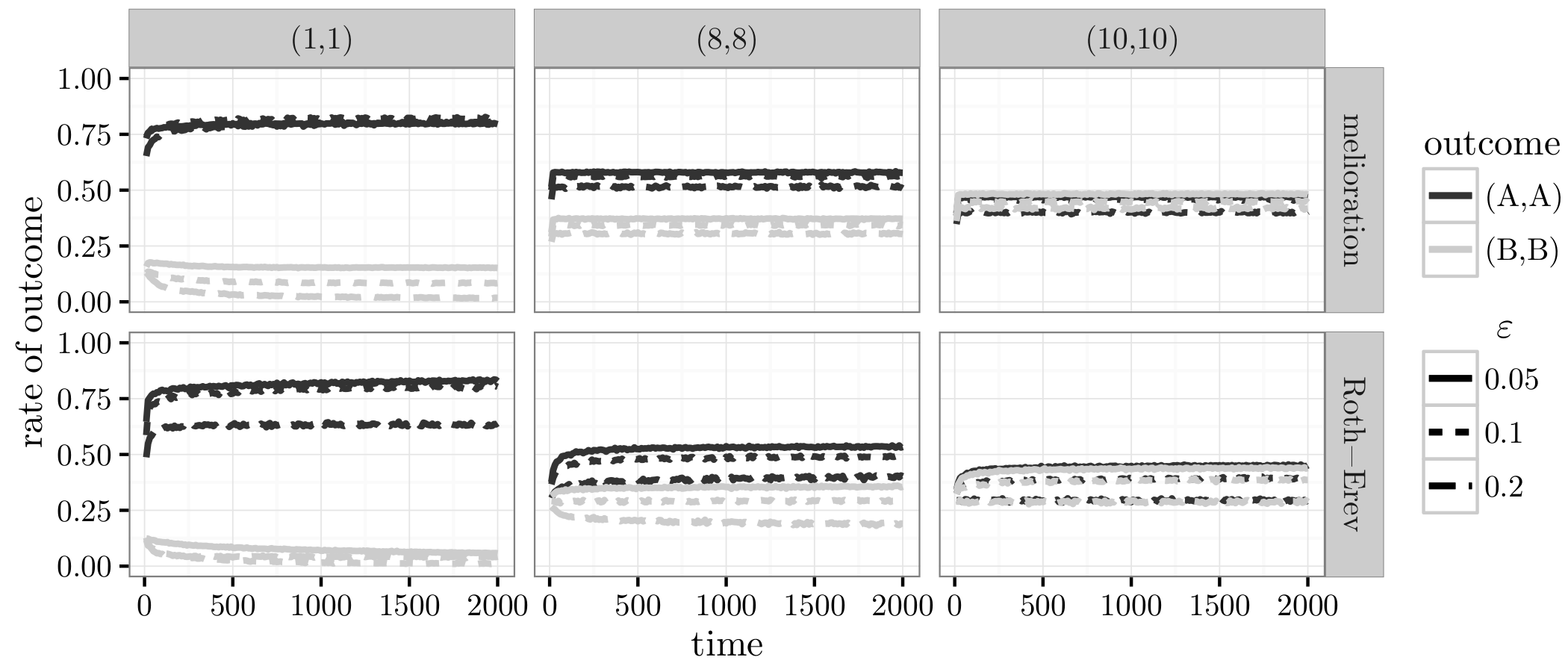

Supplement: S4 Fig — The rates of the outcomes (A,A) and (B,B) in the game of Fig 5 with different rewards for (B,B). (TIFF) [file pone.0166708.s004.tiff]

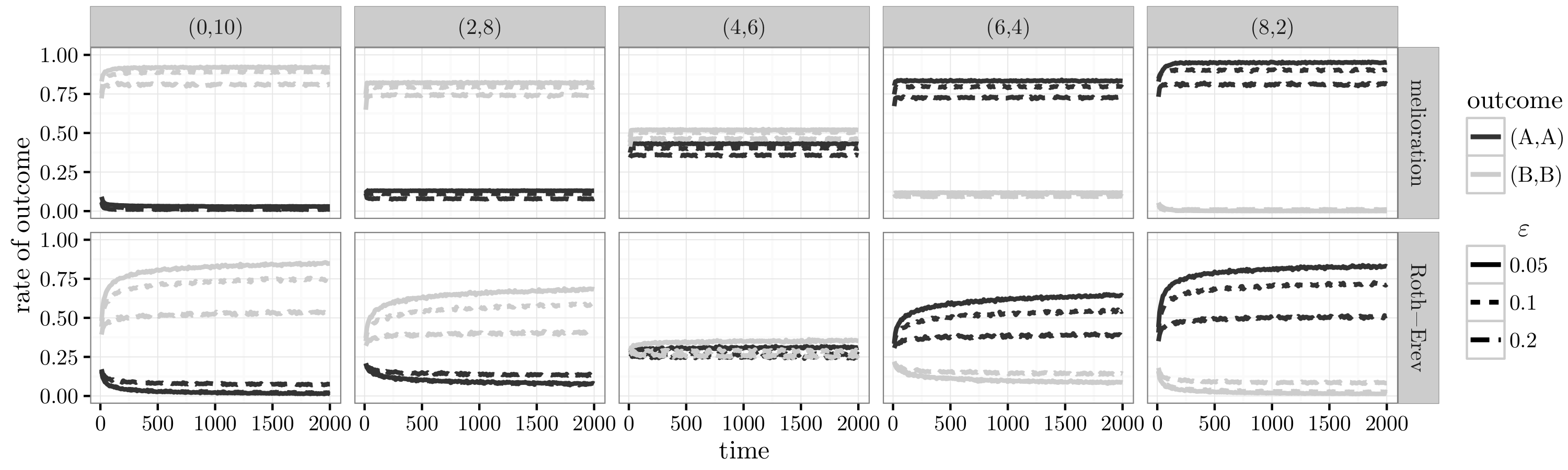

Supplement: S5 Fig — The rates of the outcomes (A,A) and (B,B) in the game of Fig 7 with different rewards (a,b). (TIFF) [file pone.0166708.s005.tiff]

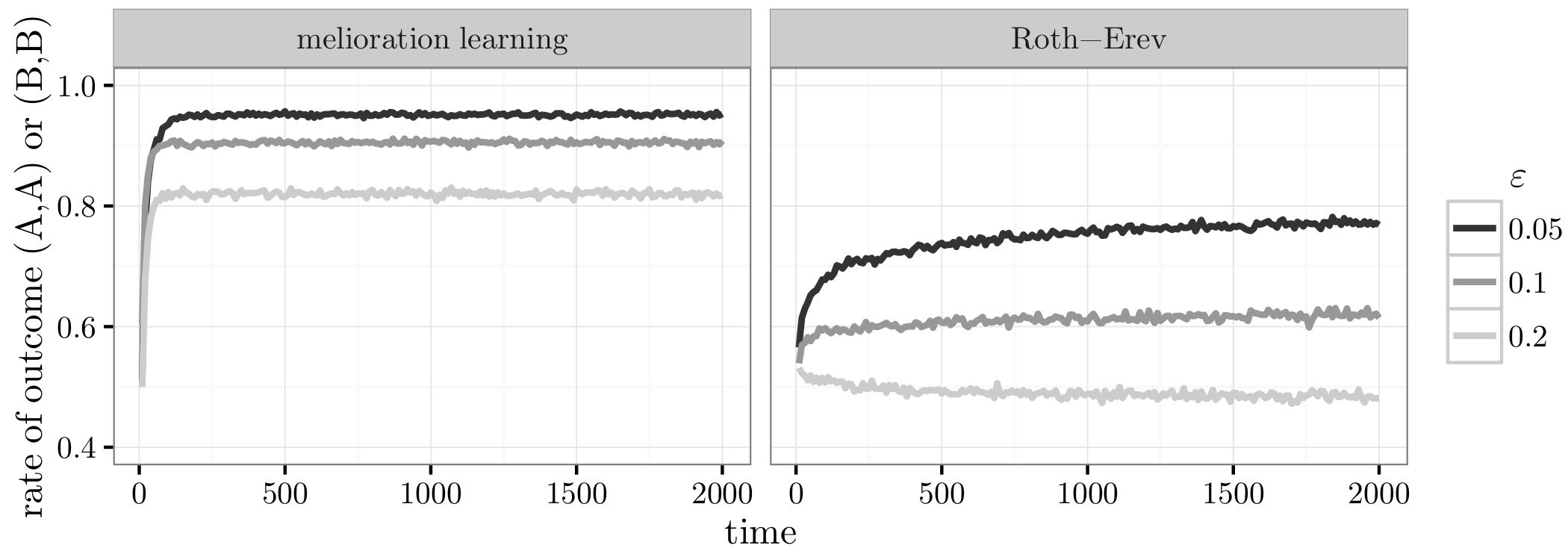

Supplement: S6 Fig — The rate of outcome (A,A) or (B,B) in the first game of Fig 8. (TIFF) [file pone.0166708.s006.tiff]

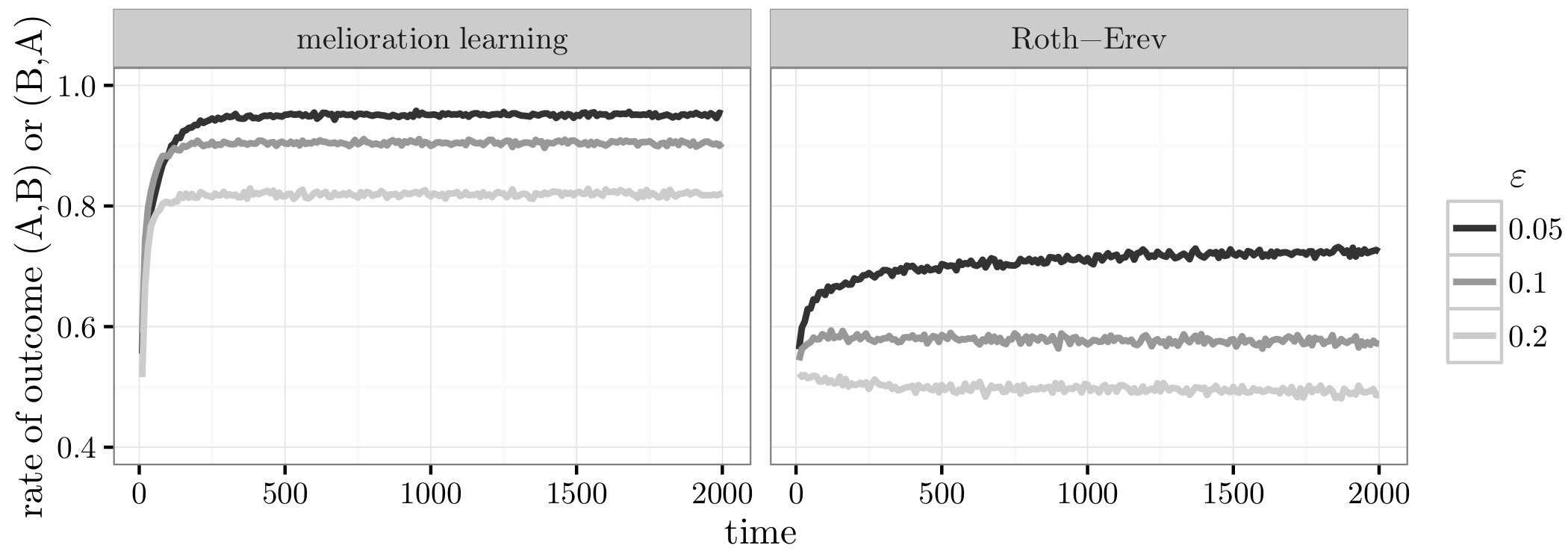

Supplement: S7 Fig — The rate of outcome (A,B) or (B,A) in the second game of Fig 8. (TIFF) [file pone.0166708.s007.tiff]

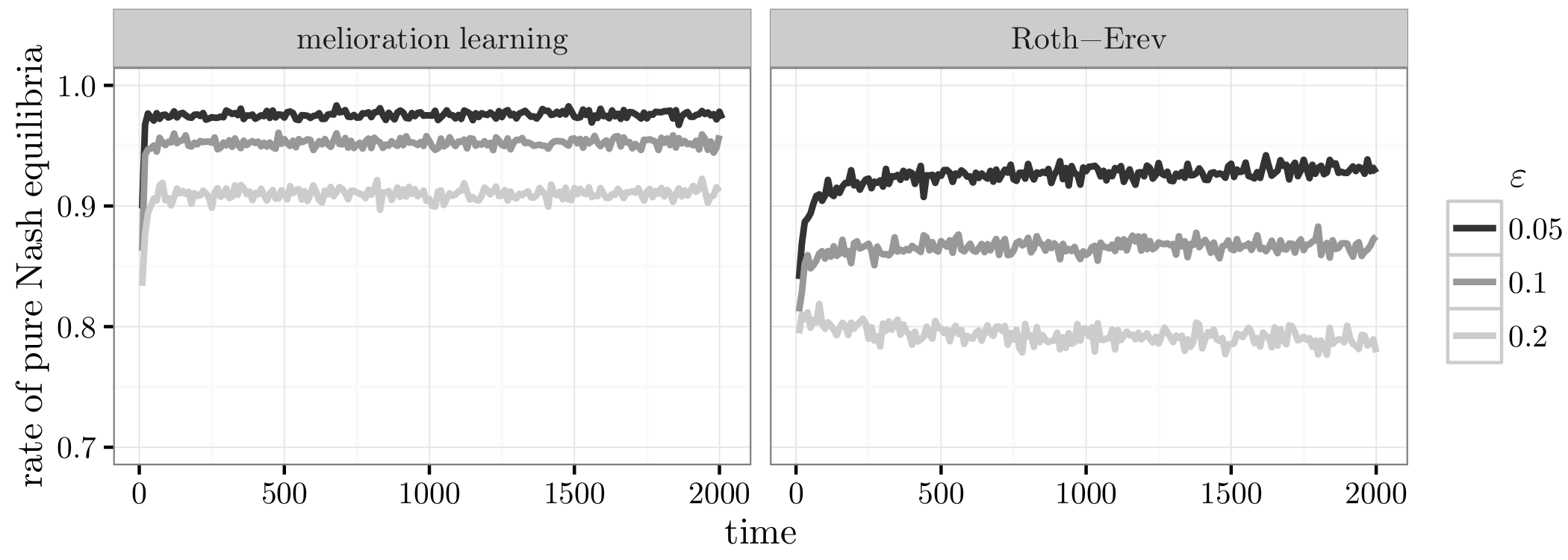

Supplement: S8 Fig — The rate of pure Nash equilibria in the game of Fig 9. (TIFF) [file pone.0166708.s008.tiff]

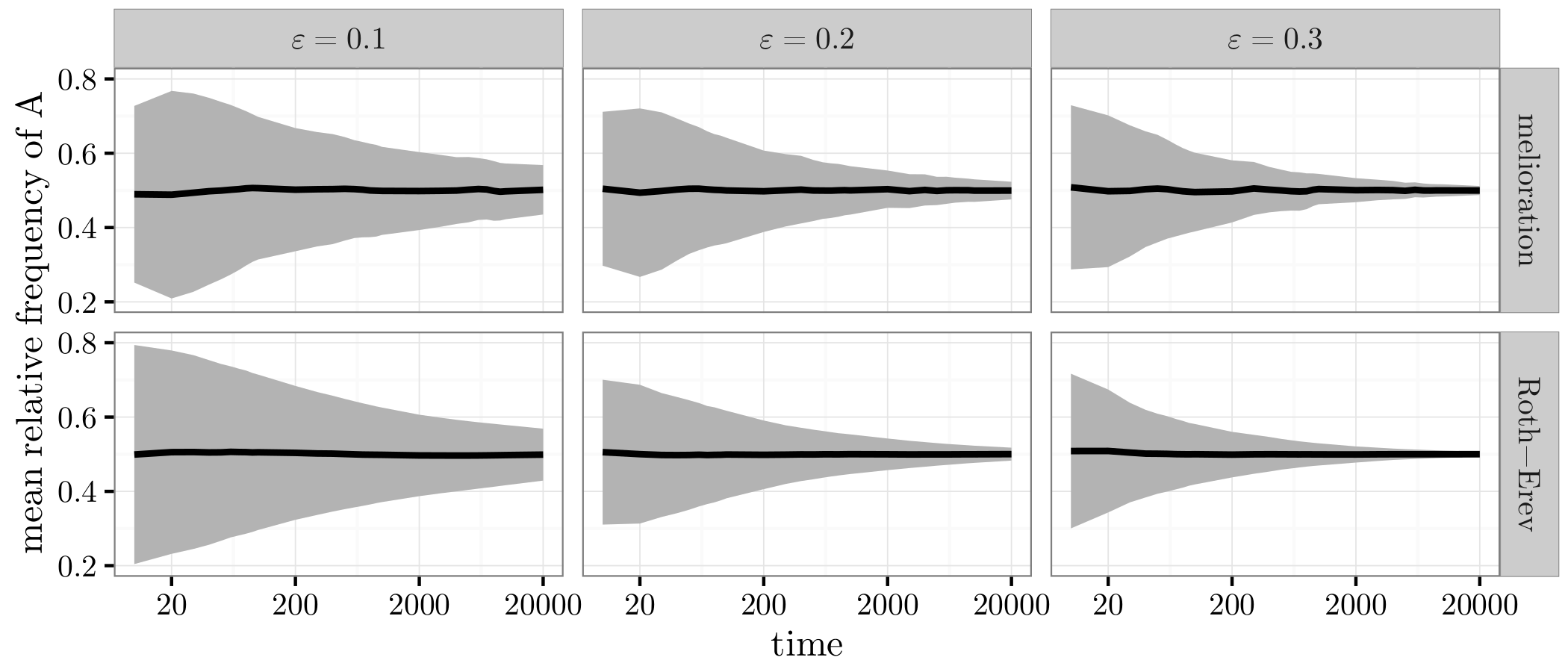

Supplement: S9 Fig — The mean relative frequency of alternative A in the game of Fig 10. The ribbon indicates the standard deviation. The relative frequencies at a time t were calculated for the period from the start of the simulation until time point t. (TIFF) [file pone.0166708.s009.tiff]

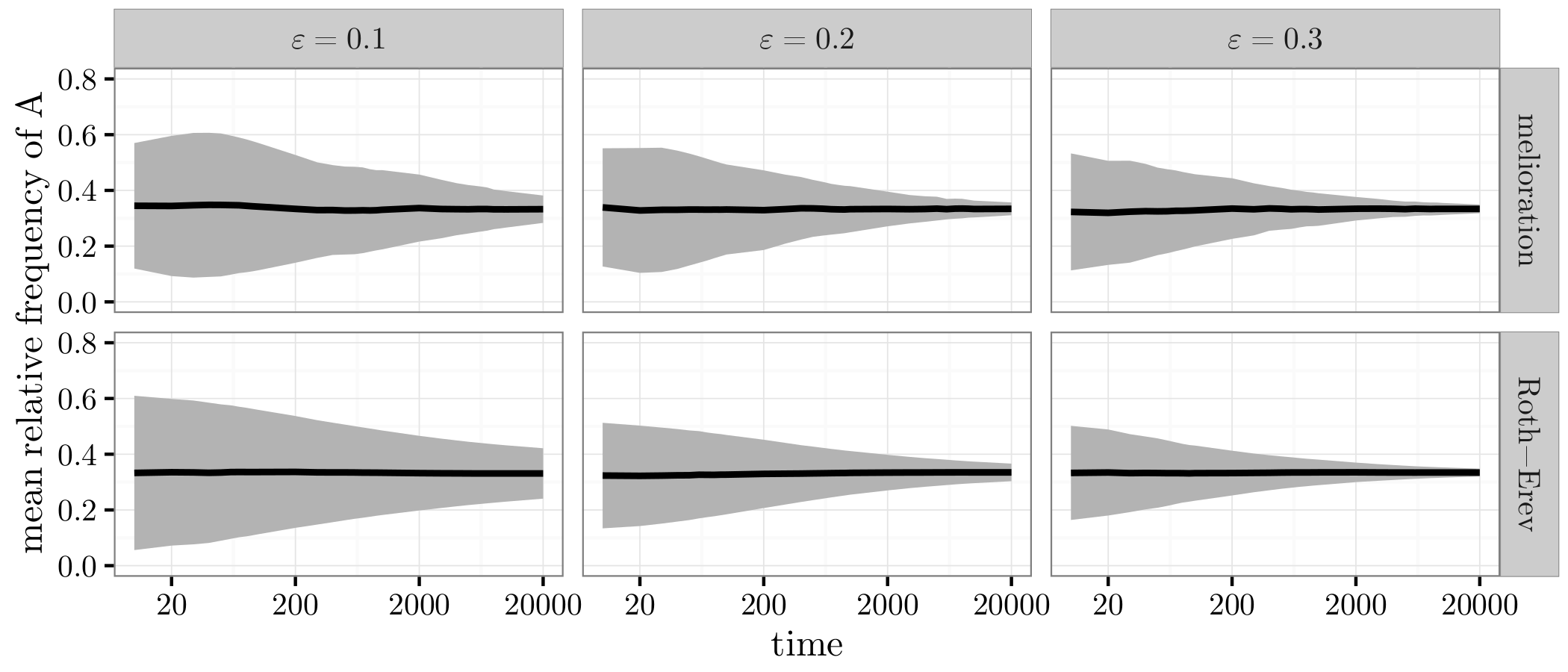

Supplement: S10 Fig — The mean relative frequency of alternative A in the game of Fig 11. The ribbon indicates the standard deviation. The relative frequencies at a time t were calculated for the period from the start of the simulation until time point t. (TIFF) [file pone.0166708.s010.tiff]

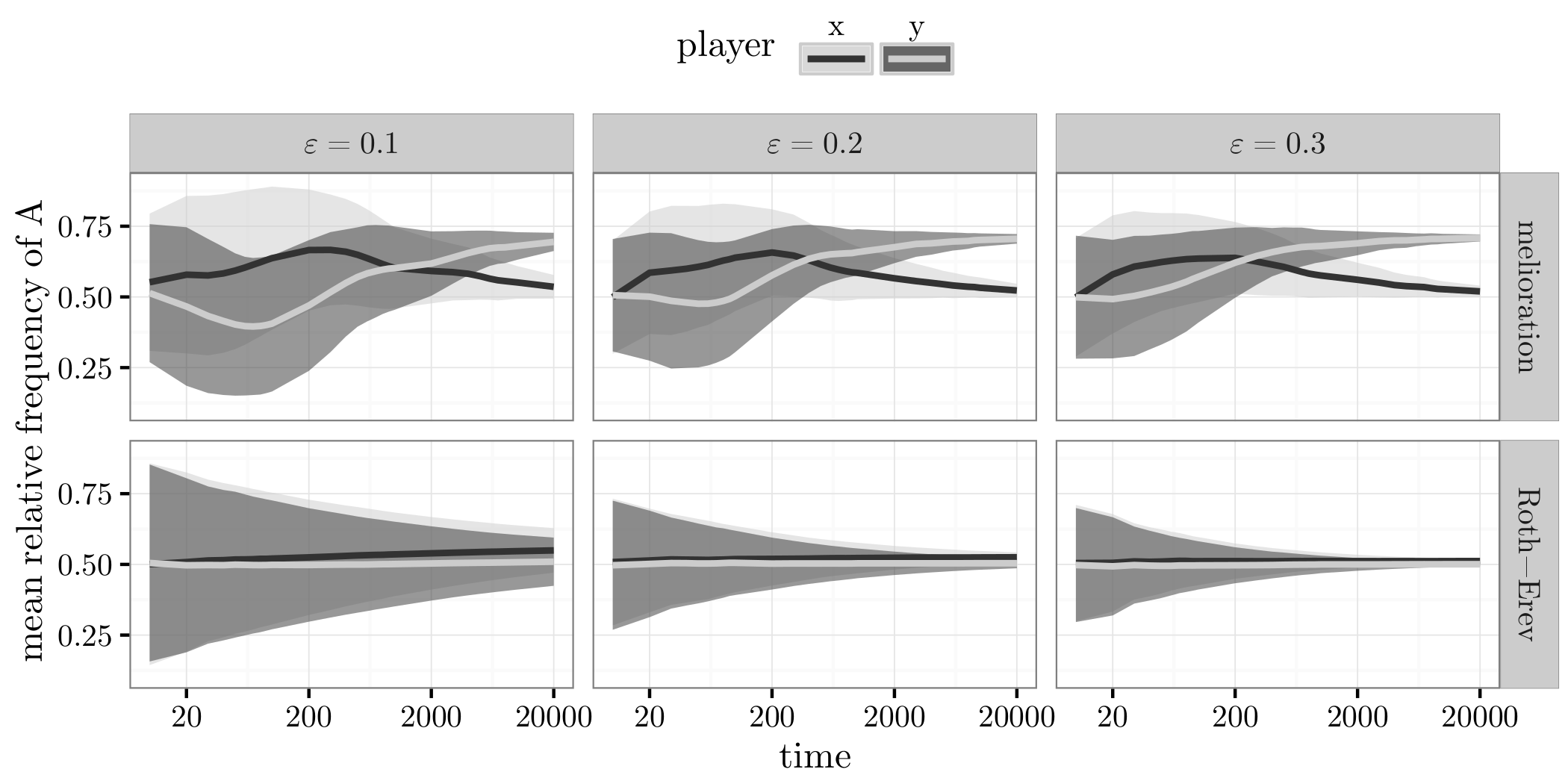

Supplement: S11 Fig — The mean relative frequency of alternative A in the game of Fig 12. The ribbon indicates the standard deviation. The relative frequencies at a time t were calculated for the period from the start of the simulation until time point t. (TIFF) [file pone.0166708.s011.tiff]
